# Health and access to healthcare in homeless people: Protocol for a mixed-methods study

**DOI:** 10.1097/MD.0000000000028816

**Published:** 2022-02-18

**Authors:** Miguel A. Bedmar, Miquel Bennasar-Veny, Berta Artigas-Lelong, Francesca Salvà-Mut, Joan Pou, Laura Capitán-Moyano, Mauro García-Toro, Aina M. Yáñez

**Affiliations:** a Research Group on Global Health & Human Development, University of the Balearic Islands, Palma, Spain; b Department of Nursing and Physiotherapy, University of the Balearic Islands, Palma, Spain; c Research Group on Global Health & Lifestyle, Health Research Institute of the Balearic Islands (IdISBa), Palma, Spain; d CIBER de Epidemiología y Salud Pública (CIBERESP), Madrid, Spain.; e Department of Applied Pedagogy and Education Psychology, Institute for Educational Research and Innovation, University of the Balearic Islands, Palma, Spain; f Primary Health Care, Balearic Islands Health Services, Palma, Spain; g Research Group on Mental Disorders of High Prevalence (TRAMAP), Research Institute of Health Sciences (IUNICS), University of the Balearic Islands, Palma, Illes Balears, Spain; h Network for Research on Chronicity, Primary Care, and Health Promotion (RICAPPS), Palma, Spain.

**Keywords:** health, healthcare access, homeless persons, inequalities in health

## Abstract

**Background:**

Homelessness is a more complex problem than the simple lack of a place to live. Homeless people (HP) often suffer from poor health and premature death due to their limited access healthcare, and are also deprived of basic human and social rights. The study protocol described here aims to evaluate the complex relationship between homelessness and health, and identify the barriers and facilitators that impact access to healthcare by HP.

**Methods:**

This is a mixed-methods study that uses an explanatory sequential design. The first phase will consist of a cross-sectional study of 300 HP. Specific health questionnaires will be used to obtain information on health status, challenges during the COVID-19 pandemic, self-reported use of healthcare, diagnoses and pharmacologic treatments, substance abuse (DAST-10), diet quality (IASE), depression (PHQ-9), and human basic needs and social support (SSQ-6). The second phase will be a qualitative study of HP using the “life story” technique with purposive sampling. We will determine the effects of different personal, family, and structural factors on the life and health status of participants. The interviews will be structured and defined using Nussbaum's capability approach.

**Discussion:**

It is well-known that HP experience poor health and premature death, but more information is needed about the influence of the different specific social determinants of these outcomes and about the barriers and facilitators that affect the access of HP to healthcare. The results of this mixed methods study will help to develop global health strategies that improve the health and access to healthcare in HP.

## 1 Introduction

Homeless people (HP) suffer greater social exclusion and health inequities than many other vulnerable groups.^[1]^ Homelessness is more complex than the simple absence of a place to live, and is a major challenge for policy makers worldwide who attempt to address health and social inequities. Moreover, HP are commonly deprived of their full basic human and social rights. Homelessness should be considered within the broader context of socioeconomic factors that affect health inequalities and social justice.^[2]^ Homelessness is a consequence of the interactions of individual and structural factors,^[3]^ and is affected by changes in family structure, migration, and the rising cost of housing. This leads to a lack of emotional, physical, and legal security in HP.^[4]^ A goal of the United Nations 2030 Agenda for Sustainable Development is the commitment by different counties to provide access to adequate, safe, and affordable housing, and basic services for all people.^[1]^ Consequently, a major public and social policy challenge is to understand the phenomenon of homelessness through the perspectives of global health,^[5]^ human dignity, and social justice.^[^2^,^^6^^]^

The European Federation of National Organizations Working with Homeless defines homelessness as “people living in a place of habitation (…) that is below a minimum adequacy standard, and lacking access to adequate housing”. However, there is no international consensus on the definition of homelessness, and previous studies used different approaches to operationalize the definition of homelessness.^[7]^

Homelessness and housing exclusion have become increasing problems worldwide over the last decade.^[^8^–^11^]^ The international financial crisis during 2007 and 2008 generated more inequalities in many societies, especially by creating new barriers to housing because of increased poverty.^[12]^ In the last ten years, the percentage of HP increased by 70% in Europe, and Spain currently has an estimated 33,275 HP.^[^11^,^^12^^]^ Analyses of the sociodemographic characteristics of HP indicated that many of them are middle-aged men who have experienced chronic homelessness.^[13]^ However, unemployment, migration, aging of the population, and changes in family structure have led to changes in the sociodemographic characteristics of HP. Thus, homelessness is now more common among foreigners, the elderly, and women.^[^11^,^^14^^]^ In addition, the COVID-19 pandemic contributed to increased unemployment, poverty, and vulnerability in many people, and these could contribute to an increased number of HP.

Homelessness and poor health are closely related.^[^15^,^^16^^]^ Access to housing means more than staying warm or feeling protected; it also provides privacy, dignity, and the sense of social inclusion. Based on the capability approach first described by Sen and Nussbaum, living without a home impacts health and human development.^[5]^ The life expectancy of HP is about 30 years less than those with homes, and HP who remain homeless have a significantly lower 10-year life expectancy.^[9]^ Moreover, HP have a high risk for many adverse health outcomes, such as communicable diseases and foot infections, and often have low adherence to necessary pharmacological treatments. Some studies reported that about 60% to 80% of HP have a mental health condition, drug dependence, or a concurrent disorder.^[^15^,^^17^^]^ Notably, 45% of HP have a high risk of suicide and 9% to 29% have attempted suicide.^[18]^ HP also suffer from other serious chronic diseases, such as diabetes and hypertension; although the prevalences of these conditions are similar in HP and the general population, HP are more likely to develop complications and disabilities.^[19]^ A study of HP in the U.S. reported high prevalences of tuberculosis (0.2%–7.7%), hepatitis C (3.9%–36.2%), and HIV-positivity (0.3%–21.1%).^[20]^ Other research reported that 7.3% to 39.9% of HP suffer from sexually transmitted infections (chlamydia, gonorrhea, or hepatitis C).^[21]^ However, differences among tests, screening, contexts, and sexual behaviors among studies make it difficult to compare different populations.^[^20^–^22^]^

Access to healthcare in countries without universal health coverage is affected by individual financial status,^[23]^ and HP and those living in poverty in these countries therefore face a significant burden. HP from Canada, countries in the European Union, and other countries with Universal Health Coverage may nonetheless experience limited access to healthcare because of competing needs and priorities, limited physical access to healthcare, difficulties in contacting needed healthcare providers, unreliable access to medications, discrimination, and inflexibility of the healthcare system.^[^8^,^^17^^,^^24^^,^^25^^]^ Similarly, HP may ignore basic health recommendations because their needs for food and shelter are more urgent.

HP account for more hospital admissions and longer hospital stays than people from the general population who have the same conditions.^[^17^,^^26^^,^^27^^]^ However, HP have fewer visits to general practitioners than people with homes.^[28]^ For example, a study in England showed that 60% of HP did not visit a primary health center during the 5 previous years.^[29]^

The current COVID-19 pandemic increased the vulnerability and health risks of HP.^[^30^,^^31^^]^ The pandemic-related factors responsible for this are the disease itself and the consequences of the pandemic, such as internal displacement (possibly leading to greater exposure to infected individuals), lack of access to toilets and showers, nutritional deficiencies, physical and mental health problems (possibly leading to reduced awareness of different risks), and more limited access to healthcare services.^[^30^,^^32^^,^^33^^]^ Thus, HP may have become more vulnerable less protected than before the pandemic, and may therefore present a greater risk for themselves and the community. A previous study of the SARS epidemic in 2003 noted that many HP were infected because of deficiencies in communication and coordination among politicians, social services, healthcare services, and non-governmental organizations (NGOs).^[34]^ This study also identified 2 major problems faced by HP: a high level of underdiagnosis, and difficulties in finding places for healthy and safe confinement. Some important institutions, such as the U.S. Centers for Disease Control and Prevention, recommended some significant new public health interventions during the COVID-19 pandemic: massive screening for COVID-19 in HP, reducing the overcrowding of shelters, providing different facilities for HP depending on symptoms and PCR test results, and increasing access to basic services.^[^35^–^37^]^ Therefore, meaningful and interdisciplinary interventions are needed to address the vulnerability of HP to health problems. Moreover, because of the social and financial disruptions caused by the COVID-19 pandemic, people who previously faced other types of residential exclusion may now be homeless.

Homelessness is a multidimensional and complex problem that has a direct impact on health status and access to healthcare.^[38]^ Furthermore, the interactions between homelessness and health are bi-directional and under-studied, and there have been very few qualitative studies.^[6]^

## 2 Objectives

A mixed-methods approach will be applied to analyze the relationship between homelessness and health. Based on the results, policies and interventions that aim to improve the health and access to healthcare of HP will be proposed.

The specific objectives of the study are:

1. To describe the health status of HP using a social determinants of health framework.
2. To identify barriers and facilitators that affect access to healthcare by HP.
3. To identify and examine the life stories of HP to better understand the complex relationships between health status and homelessness.
4. To explore knowledge, attitudes, and practices of HP during the COVID-19 pandemic.

## 3 Methods

This is a mixed-methods study that uses an explanatory sequential design.^[39]^ During the first phase, we will perform quantitative data collection, and during the second phase we will perform a qualitative study to clarify, interpret, and describe the quantitative results using a complementary approach.

### 3.1 Quantitative phase

#### 3.1.1 Sample size and participants

We aim to examine all potential participants (approximately 300 HP) in Palma de Mallorca, according to data from 2019.^[40]^ The inclusion criteria will be:

1. age of at least 18 years;
2. living in Palma and meeting the European Typology of Homelessness and Housing Exclusion (ETHOS) classification (5); and
3. agreeing to participate in the study and signing the informed consent document. For operational purposes, we define HP as those who, during last year, lived in the streets, or other public areas, or in an abandoned building, or in place that does not meet minimum conditions for habitability (i.e., without power or water supplies) and excludes HP.

This is similar to the definition used previously.^[24]^ The exclusion criteria will be:

1. staying overnight in a private or municipal shelter for more than 3 months during the previous year;
2. having an acute episode of mental disease or being under the influence of alcohol or any drug during recruitment.

#### 3.1.2 Data collection

We will collaborate with 2 NGOs that work on specific programs with HP in the city of Palma. Their facilities will be used for the individual interviews and blood tests. Moreover, a worker from the NGOs will collaborate in contacting potential participants and arranging appointments. A nurse from our research group will collect data using a questionnaire and will perform blood tests. The estimated time for both activities is 30 to 40 minutes. Later, another nurse will check the electronic health records of all participants. We will give financial compensation to all participating HP.

A questionnaire structured in 4 blocks will be administered to assess the following:

1. sociodemographic characteristics (including age, sex, nationality, educational level, last occupation, receipt of public subsidies, time of homelessness, and shelter visits during last year);
2. challenges experienced during the COVID-19 pandemic, especially during the confinement period (March to June of 2020), difficulties in finding essential resources, and compliance with sanitary measures (use of surgical masks, hand washing, and social distancing);
3. self-reported use of healthcare services, diagnoses and pharmacologic treatments, and assessments of substance abuse (DAST-10), diet quality (IASE), and depression symptoms (PHQ-9);
4. human basic needs assessment, adapted from Virginia Henderson's Needs theory.^[41]^ We will also ask participants about feeding, safety, hygiene, and sleep, and will assess social support using the SSQ-6 questionnaire.^[42]^

##### 3.1.2.1 Drug Abuse Screening Test

The DAST is a questionnaire used to identify drug abuse in adults, and a short form (DAST-10) was validated for the Spanish population.^[43]^ The possible answers for each item are “yes” or “no,” the maximum score is 10 points, and a score of 3 or more is used to define substance abuse.

##### 3.1.2.2 Healthy feeding index

The IASE measures 10 items to determine diet quality, and was validated for the Spanish population.^[44]^ Nine measures are related to food groups and one is about diet variety. The score for each item is based on the adequacy of consuming the appropriate amount of each food group. Each item has a score between 0 and 10, and the total score is the sum of all ten items (range: 0–100 points). The different classifications are “healthy diet” (>80 points), “diet that needs changes” (50–80 points), and “unhealthy diet” (<50 points).

##### 3.1.2.3 Patient Health Questionnaire

The PHQ-9 is a tool used to diagnose and assess the severity of depression, and was validated for the Spanish population.^[45]^ The 9 items ask about symptoms during the previous 2 weeks. Each answer is scored using a Likert scale, and varies from 0 to 3 points (total range: 0–27). The total score is used to characterize a participant as having “no depressive symptoms” (0–4), “mild depressive symptoms” (5–9), “moderate depressive symptoms” (10–14), “moderately severe depressive symptoms” (15–19), or “severe depressive symptoms” (20–27). A previous study of HP established a cut-off of 10 points.^[46]^

##### 3.1.2.4 Social Support Questionnaire

The SSQ-6 is a 6-item questionnaire derived from a longer questionnaire (SSQ) that assesses perceived social support, and was validated for the Spanish population.^[42]^ Each item asks the study participant about different occurrences that lead to stress or the need for assistance, the number of people that can be relied upon during that time, and their satisfaction with the perceived social support.

##### 3.1.2.5 Blood test

Blood will be collected for serology testing of SARS-CoV-2, HIV, syphilis, and hepatitis B and C, and for measurements of glycosylated hemoglobin. Blood samples will be managed following the usual procedures for primary health care facilities, and analyzed by the referral hospital (Hospital Universitario Son Espases).

##### 3.1.2.6 Health record review

We will also analyze the electronic health records and examine information about the number of visits to different services during the previous 2 years (Primary Healthcare, Primary Healthcare Emergency Department, Addictive Behaviors Unit, Mental Health Unit, Oral Health Unit), and will also assess the use of hospital outpatient consulting, hospitalization, and admission to emergency departments. All diagnoses and pharmacologic treatments during the previous year will also be assessed.

#### 3.1.3 Data analysis

The survey will be prepared and administered using the TeleForm program (Cardiff Software, Vista, CA, USA), which allows automated data entry and subsequent verification. All continuous variables will be presented as means and standard deviations or medians and interquartile ranges, depending on variable distribution. The categorical variables will be presented as absolute numbers and relative frequencies. We will calculate the prevalences of different diagnoses in HP using percentages and a 95% CIs. The Chi-Squared test will be used to compare categorical variables, and Student's *t* test to compare continuous variables. All statistical analyses will be performed using Statistical Package for Social Science software (SPSS) version 24 (IBM, NY, IL) and a *P* value below .05 will be considered significant.

### 3.2 Qualitative phase

#### 3.2.1 Theoretical-methodological framework

We will use a critical theory framework and an ethnosociological approach to examine the relationship of homelessness and health. This perspective considers homelessness to be a consequence of the power relationships in society and in a given context. We will examine the social determinants of health using Nussbaum's capability approach.^[^2^,^^38^^]^ This framework will be used to analyze and examine the relationships of human dignity and social justice with homelessness and health.^[47]^

Moderate inductive and deductive logic will be used in a predominantly qualitative approach that is developed from a quantitative approach.^[^48^,^^49^^]^ This will highlight different experiences and the interactions of homelessness and health in an effort to characterize them as inseparable. This phenomenological description places the researchers and participants at the convergence of subjective testimony and social reality.^[50]^

#### 3.2.2 Sampling methods and participants

A purposive sampling design will be used in an effort to achieve high variability among the enrolled HP. Thus, the sample will be stratified by sex, age, sexual orientation, duration of homelessness, sleeping location, and administrative status. The saturation and representativeness of the data will limit the number of participants to a manageable level.

#### 3.2.3 Data collection

Semi-structured interviews will be performed. More specifically, “life stories” will be used for a biographical analysis of an individual's life trajectory before and after homelessness.^[^51^,^^52^^]^ This technique will allow us to explore and know how different personal, family, and structural factors contributed to homelessness and health of HP. The interviews will be structured and defined through Nussbaum's capability approach^[2]^ and will address the following blocks:

1. current health situation: COVID-19 pandemic, health, and access to healthcare services;
2. circumstances that led to homelessness and previous way of life; and
3. current situation and life prospects.

The first block will allow concise analysis of the experience during the COVID-19 pandemic. The second block will review the life trajectory that defines an individual's current situation in terms of structural, personal, and emotional aspects, and will allow examination of the relationship between homelessness and health. The third block will assess the individual's perspective and will record hopes, life prospects, and perception of control over the environment.

We expect to perform 15 to 20 interviews at a location agreed upon with the participant. Interviews will be administered until data saturation is achieved. The participants will be contacted directly by telephone or through NGOs, each interview will last 60 to 90 min, and the participants will be remunerated. A second meeting with each participant will be scheduled when necessary. Interviews will be audio-recorded for subsequent transcription. During the interview, researchers will also record notes in a field diary.

#### 3.2.4 Data analysis

An analysis of the thematic and comprehensive content of the corpus of life stories will be performed. Given the study design, this will be a cumulative analysis of the different life stories.

An analysis of all transcripts will be performed by reading the life story of each participant. The richest life story, in terms of the description of the experience, will be selected and deconstructed into units and themes. Furthermore, a thematic analysis will be used to identify the transversal elements of the different stories.

After the topics are categorized, we will perform a comprehensive analysis of the corpus of stories. We will also analyze life trajectories to identify different factors involved in the relationship between homelessness and health status. Thus, a diachronic analysis will be used to establish a connection of the periods before and after homelessness and to determine how this connection developed over time. The fieldwork notes will be used to complement the transcripts by providing records of non-verbal cues, context, and/or emotions.

To guarantee methodological rigor, data triangulation will be used to achieve saturation. Thus, 2 researchers will perform these methods using reflection and discussion. Then, a third researcher will discuss the results with health and social professionals. ATLAS.ti version 9.0 will be used to manage these data.

### 3.3 Integrating quantitative and qualitative data

First, we will integrate the results from quantitative data to design the qualitative interviews. In particular, we will select specific issues that were not well described in the quantitative phase for inclusion in the qualitative phase. Second, we will triangulate the quantitative and qualitative data to improve our understanding of homelessness and health, will increase the validity of the overall findings by using different perspectives, and will capture convergence and divergence using clear and reproducible rules.^[53]^ Finally, we will discuss the main findings from both designs by integrating the qualitative and quantitative data.

## 4 Discussion

This mixed design study aims to identify the complex relationship between homelessness and health status. HP are extremely vulnerable to health problems, and our study will analyze their health, health needs, access to healthcare services, life trajectories, and recent challenges during the COVID-19 pandemic. We hypothesize that government restrictions will be identified as a threat to the capability for life, health, and bodily integrity of HP. As Nussbaum stated, capabilities are simplified to assure survival during a crisis.^[5]^

Reducing homelessness, eradicating poverty, reducing inequality, ensuring healthy living, and fostering well-being are some of the goals of the United Nations 2030 Agenda for Sustainable Development, goals to which all countries should aspire. However, the perspectives and experiences of HP are often not considered when developing policies related to healthcare and social care.^[^1^,^^54^^]^ Thus, this mixed-methods study will provide a global vision from the perspective of HP.

Furthermore, this study will examine the experiences of HP during the confinement period of the COVID-19 pandemic, and may be useful for establishing a guide for future epidemics or pandemics. Additionally, information about access and utilization of healthcare by HP and their health-related needs may be useful for planning adequate healthcare and social resources for this population.

It should be noted that difficulties in contacting and recruiting certain HP could bias our study, because we may miss the most vulnerable and extreme cases. Thus, we will work with 2 NGOs that regularly assist HP in an effort to reduce this selection bias. We will try to establish a relationship of trust between the interviewer and homeless participants to obtain the most reliable data possible. Finally, although this will be an evaluation of HP in Spanish people, our results may be applicable in other contexts, countries, and activities.

In conclusion, this project will provide an opportunity to highlight the critical situations that HP currently face, and we will strongly advocate improvements in the health, healthcare, and social justice for HP.

## Acknowledgments

We would like to thank the essential help received from *Medicos del Mundo* and *Creu Roja*.

## Author contributions

**Conceptualization:** Miguel A. Bedmar, Miquel Bennasar-Veny, Berta Artigas-Lelong, Aina M. Yáñez.

**Data curation:** Miguel A. Bedmar, Joan Pou, Laura Capitán-Moyano.

**Investigation:** Miguel A. Bedmar, Miquel Bennasar-Veny, Mauro García-Toro, Aina M. Yáñez.

**Methodology:** Miguel A. Bedmar, Miquel Bennasar-Veny, Berta Artigas-Lelong, Aina M. Yáñez.

**Supervision:** Miquel Bennasar-Veny, Aina M. Yáñez.

**Validation:** Francesca Salvà-Mut, Mauro García-Toro.

**Writing – original draft:** Miguel A. Bedmar, Laura Capitán-Moyano.

**Writing – review & editing:** Miquel Bennasar-Veny, Berta Artigas-Lelong, Francesca Salvà-Mut, Joan Pou, Mauro García-Toro, Aina M. Yáñez.
